# Numerical Simulation Analysis of the Bending Performance of Straw–Concrete Combined Floor Slabs

**DOI:** 10.3390/ma18051070

**Published:** 2025-02-27

**Authors:** Shuoran Li, Yufei Chen, Haibiao Wang, Jida Liu, Lin Li, Jingyi Liu

**Affiliations:** School of Civil Engineering and Transportation, Northeast Forestry University, Harbin 150040, China; nefulsr@nefu.edu.cn (S.L.); 20222124544@nefu.edu.cn (Y.C.); 2022212464@nefu.edu.cn (J.L.); 2022222486@nefu.edu.cn (L.L.); 2024224869@nefu.edu.cn (J.L.)

**Keywords:** combined floor slabs, straw, bending resistance, finite element analysis

## Abstract

Straw–concrete combined floor slabs consist of straw boards, shear-resistant connectors, and concrete slabs. These slabs offer various advantages over traditional reinforced concrete slabs due to the straw boards’ properties of excellent insulation and sound absorption. Research using ABAQUS software created 15 composite floor models to study the impact of connection methods, bond strength, connector spacing, and thickness of straw and concrete on the flexural performance. Results indicated that the composite floor slab with adhesive bonding had a 7.34% and 17.34% higher load-carrying capacity than the bolt-connected and self-tapping screw-connected composite floor slabs, respectively. Increasing bond strength from 40 MPa to 60 MPa improved the load-carrying capacity of self-tapping nail-connected slabs by 80.84%. Connector spacing negatively correlated with slab capacity, while increasing the thickness of straw boards or concrete slabs enhanced the ultimate load-carrying capacity, with the latter having a more significant effect. Midspan deflection and flexural capacity were calculated using the converted cross-section method and static calculation formulas, with theoretical and simulated values showing good agreement, offering guidance for engineering applications.

## 1. Introduction

Straw is an ideal choice for new green building materials due to its rich resource reserves, environmental friendliness, and low production costs, which can meet the growing demand for raw materials in the construction industry [1,2]. Currently, most of the existing straw boards are made of corn straw, wheat straw, and MDI (4,4′-diphenylmethane diisocyanate), with zero formaldehyde content and mechanical properties almost comparable to those of synthetic plywood. These straw boards have been partially applied to the furniture industry and can be used for decorative panels, sound-absorbing panels, and thermal insulation panels [3,4]. The use of straw boards to replace wood is completely feasible, not only to protect forest resources but also to solve the problem of straw storage and disposal [5,6].

Alam et al. [7] used wheat straw, rice straw, and pineapple crown waste as raw materials for preparing straw boards in order to improve the mechanical properties of the boards. A research team has already prepared concrete using straw ash as a partial substitute for cement to improve the durability, fluidity, and mechanical properties of the material [8,9,10]. Researchers have also incorporated different types and contents of straw fibers into concrete as reinforcing materials and have achieved more results related to the physical properties, mechanical properties, and thermal insulation properties of the composite materials [11,12,13].

Hu Mingming [14] conducted bending tests and finite element simulations on light steel–straw concrete composite floor slabs, proving that the deformation performance and failure mechanism of the composite floor slabs are similar to those of traditional one-way slabs and can be widely applied in prefabricated buildings. Zhou Xiaoqun et al. [15] investigated the damage process and working mechanism of an aerated concrete composite wall with embedded straw panels, and the results showed that the composite wall had a good effect of vibration damping and energy dissipation. Xu Z et al. [16] subjected a new type of straw fiberboard cold-formed thin-walled steel composite wall to a compression test and analyzed the factors affecting the axial compression performance. The results showed that the straw board could further improve the bearing capacity of the composite wall. U. De Maio et al. [17] analyzed the deterioration of dynamic properties of concrete structures under fracture conditions, providing an effective means for studying the cracking–debonding problem between straw boards and concrete. Xiaoyan Ding et al. [18] used ABAQUS software to establish a finite element model and combined it with the plastic damage model to simulate the seismic performance of the new type of ecological compo straw site wall structure, providing a reference for the numerical modeling of similar structures.

Currently, the research of scholars around the world is mainly divided into two categories: composite (mixing straw and concrete into composite materials) and mechanical (using straw boards as embedded or outsourced materials), while the research on straw-based combined components is scarce. In this research, a new type of straw–concrete combined floor slab was proposed. ABAQUS finite element software was used to simulate the three-point static loading test to study the effects of the connection method, bond strength, spacing of connectors, thickness of straw boards, and thickness of concrete on the bending performance of straw–concrete combined floor slabs and to investigate the possibility of replacing part of the concrete under pressure with straw boards. Combined with the results of the finite element parameter analysis, the formula for calculating the midspan deflection and flexural capacity of straw–concrete combined floor slabs was proposed to provide a reference for the engineering design of straw–concrete combined floor slabs. The results of the paper could provide a reference for the engineering application of straw panels as green building materials, promote the development of the straw industry, and broaden the use of straw panels.

## 2. Straw–Concrete Combined Floor Slab Construction

### 2.1. Material Properties

**Figure 1:**
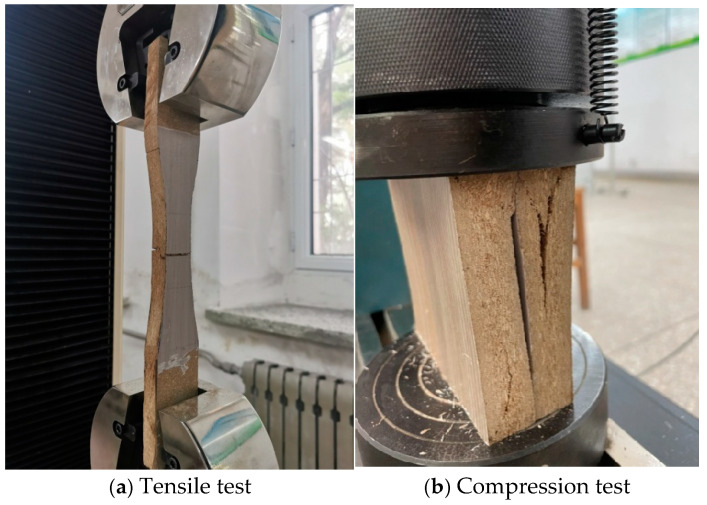
Damage phenomena for test specimens subjected to material property testing.

Straw panel structural relationship: According to the “Test Method for Mechanical Properties of Structural Artificial Panels” [19], the standard specimens of straw panels were subjected to material property testing. The damage phenomena for the tensile and compressive specimens are shown in Figure 1, and the tensile and compressive strengths and the elastic modulus are presented in Table 1.

Errors such as an uneven surface and small cracks during specimen fabrication led to the variability of the material properties in the test results. Since the straw panels are anisotropic materials with obvious fiber directions, the orthotropic anisotropic linear elasticity model was used in the simulation analysis of this study. The material properties of the straw boards showed that the compressive, tensile, and static flexural strengths of the straw boards were better than those of particleboards and density boards [20,21].

Concrete constitutive relationship: the concrete constitutive relationship, as defined in the “Code for Design of Concrete Structures” [22]. encompasses the stress–strain curve equations for uniaxial tension and compression of concrete.

**Figure 2:**
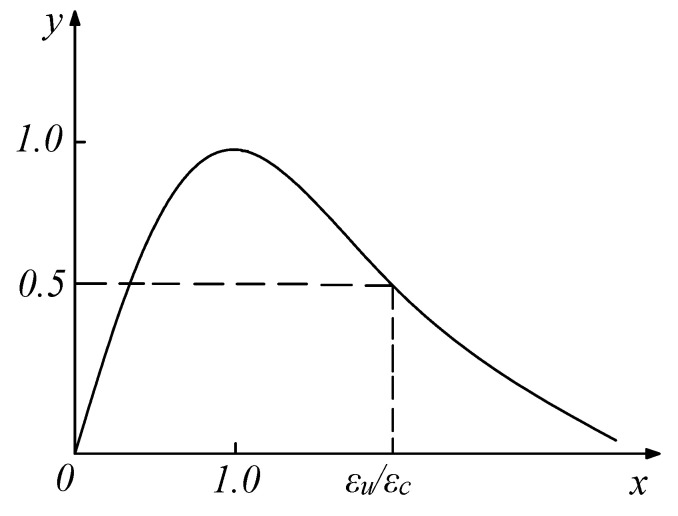
Uniaxial compression constitutive curve of concrete.

The compressive constitutive curve of concrete can be approximately divided into three segments: a linear segment in the elastic stage, an ascending segment (strengthening phase) in the elasto-plastic stage, and a descending segment (softening phase) in the plastic stage, as depicted in Figure 2.

Stress–strain curve equation for uniaxial compression of concrete:

(1) $$\sigma =\left(1-d_{c}\right)E_{c}\varepsilon$$

(2) $$d_{c}=\left\{\begin{array}{ll} 1-\frac{\rho_{c}n}{n-1+x^{n}} & x\leq 1\\ 1-\frac{\rho_{c}}{\alpha_{c}{\left(x-1\right)}^{2}+x} & x>1 \end{array}\right.$$

(3) $$\rho_{c}=\frac{f_{c,r}}{E_{c}\varepsilon_{c,r}}$$

(4) $$n=\frac{E_{c}\varepsilon_{c,r}}{E_{c}\varepsilon_{c,r}-f_{c,r}}$$

(5) $$x=\frac{\varepsilon }{\varepsilon_{c,r}}$$

In the formula, $\alpha_{c}$ is the parameter value of the descending section of the uniaxial compressive stress–strain curve of concrete; $f_{cr}$ is the uniaxial compressive strength value of concrete; $\varepsilon_{cr}$ is the peak compressive strain of concrete; $d_{c}$ is the concrete uniaxial compression damage evolution parameter.

**Figure 3:**
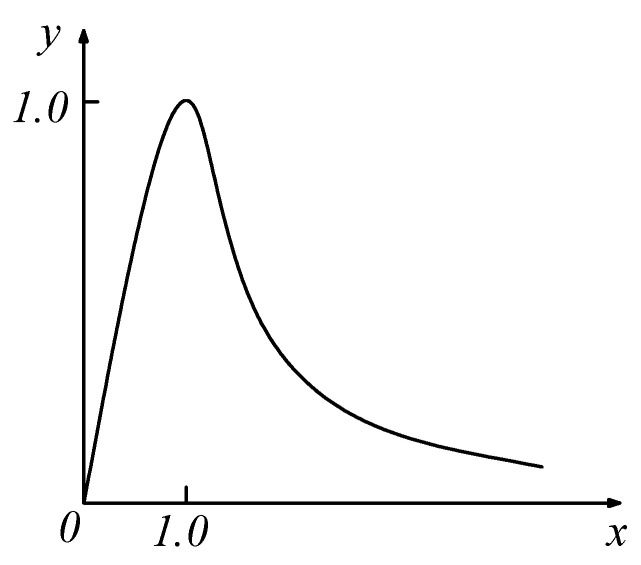
Uniaxial tensile constitutive curve of concrete.

Similar to the compressive constitutive curve, the uniaxial tensile constitutive curve of concrete is also divided into a linear segment, an ascending segment, and a descending segment, as shown in Figure 3.

Stress–strain curve equation for uniaxial tension of concrete:

(6) $$\sigma =\left(1-d_{t}\right)E_{c}\varepsilon$$

(7) $$d_{t}=\left\{\begin{array}{ll} 1-\rho_{t}\left[1.2-0.2x^{5}\right] & x\leq 1\\ 1-\frac{\rho_{t}}{\alpha_{t}+{\left(x-1\right)}^{1.7}+x} & x>1 \end{array}\right.$$

(8) $$x=\frac{\varepsilon }{\varepsilon_{t,r}}$$

(9) $$\rho_{t}=\frac{f_{t,r}}{E_{c}\varepsilon_{t,r}}$$

In the formula, $\alpha_{t}$ is the parameter value of the descending section of the uniaxial tensile stress–strain curve of concrete; $f_{t,r}$ is the uniaxial tensile strength value of concrete; $\varepsilon_{t,r}$ is the peak tensile strain of concrete; $d_{t}$ is the concrete uniaxial tensile damage evolution parameter.

**Figure 4:**
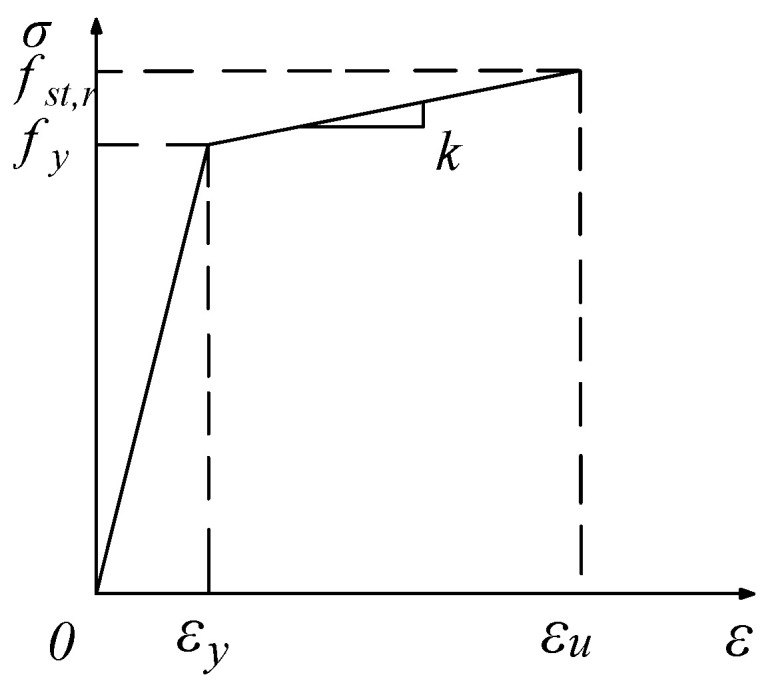
Stress–strain relationship curves of steel bars.

Rebar constitutive relationship: The constitutive relationship of the stressed steel bar refers to the elastic-plastic double diagonal model proposed in the “Code for the Design of Concrete Structures”. The stress–strain relationship curve is shown in Figure 4.

**Figure 5:**
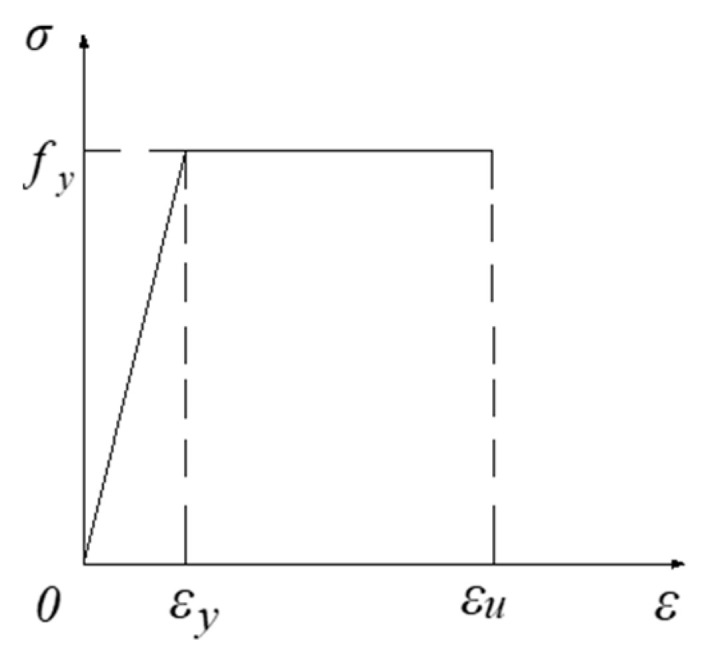
Intrinsic relationship curves of connectors.

Connector constitutive relationship: The connection types of the combined floor slab were selected as shear-resistant connectors and adhesive bonding. The shear-resistant connectors included self-tapping screws and bolts. The ideal elastic-plastic model was used for simulation. The stress–strain relationship curve is shown in Figure 5.

In Figure 4, σ represents the stress of the reinforcement, ε represents the strain of the reinforcement, $f_{y}$ represents the yield strength of the reinforcement, $\varepsilon_{y}$ is the strain corresponding to $f_{y}$, $f_{st,r}$ represents the ultimate strength of the reinforcement, $\varepsilon_{u}$ is the strain corresponding to $f_{st,r}$, and k is the slope of the reinforcement hardening section.

In Figure 5, σ represents the stress of the connector, ε represents the strain of the connector, $f_{y}$ represents the yield strength of the connector, $\varepsilon_{y}$ is the strain corresponding to $f_{y}$, and $\varepsilon_{u}$ is the maximum strain in the plastic stage of the connector.

### 2.2. Combined Floor Slab Design

**Figure 6:**
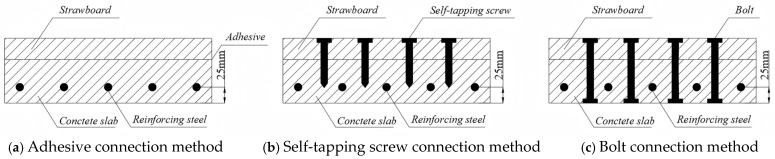
Schematic diagram of connection mode of straw–concrete composite floor slab.

During the design process of composite floor slabs, straw-based panels are employed for the upper layer, while high-strength and durable concrete panels are utilized for the lower layer to carry loads. Once the concrete has hardened, it is integrated with the straw-based panel through shear connectors or adhesive bonding. The construction connection method of the composite floor slab is illustrated in Figure 6.

Straw board, a type of panel primarily fabricated from discarded crop straw, has found extensive utilization in the construction and decoration industries. The wide-spread utilization of straw board in these industries can be attributed to its remarkable advantages, including environmental friendliness, energy efficiency, thermal insulation, sound absorption, noise reduction, as well as its lightweight nature and high strength. The composite floor slab formed by integrating straw board with concrete exhibits the following characteristics: (1) Specifically, straw board derives advantages from abundant and renewable raw materials. The resulting composite floor slab exhibits excellent elasticity and toughness, enabling it to withstand impact and vibration, which contributes to its long-term durability. (2) Moreover, the weight of straw board is significantly less than that of traditional concrete. Compared with conventional concrete floor slabs, the straw–concrete composite floor slab can save approximately 10–25% of concrete usage, effectively reducing its self-weight. Should an earthquake occur, this reduction in weight helps mitigate structural damage, making it a suitable choice for building structures in earthquake-prone regions. (3) In addition, when the straw board is installed on top of the concrete slab, it can function as a finished floor, eliminating the need for secondary decoration. However, issues related to its water absorption and durability can be addressed through strict process control and specialized treatment techniques [23]. With effective protective measures in place, the straw board, when combined with concrete, can achieve a design life of up to 50 years.

### 2.3. Simulation Scheme Design

In this paper, three shear-resistant connection methods are proposed. Overall, 15 combined floor slabs were divided into six groups of models for finite element simulation and analysis. The focus was on the investigation of the effects of connection methods, bond strength, spacing of connectors, straw board thickness, and concrete thickness on the flexural performance of the combined floor slabs.

Drawing on previous numerical models and real-world engineering cases, the predominantly adopted connection methods are self-tapping screws, bolts, and adhesive bonding. Self-tapping screws offer the advantages of convenient installation, cost-effectiveness, and reliable connection performance, making them suitable for general connections in composite floors. Bolt connections, on the other hand, demand higher connection strength and are appropriate for components of composite floors that bear relatively large loads. Adhesive bonding is capable of providing a relatively uniform bonding force, rendering it suitable for scenarios where higher requirements for appearance and structural integrity are imposed.

In composite floor structures of a similar nature, when the bonding strength falls within the range of 40 to 60 MPa, it can effectively guarantee the collaborative interaction between straw boards and concrete boards. This bonding strength level ensures that it neither causes premature separation of the two materials due to insufficient strength, which could otherwise compromise the overall structural performance, nor leads to unnecessary cost increases and construction difficulties arising from overly high strength.

The selection of the thicknesses for straw boards and concrete boards should not only satisfy the mechanical performance requirements of composite floors but also take into account the control of structural dead weight and economic considerations. Thinner straw boards and concrete boards can indeed reduce the overall weight, yet they may fail to meet the load-bearing requirements. Conversely, thicker boards can enhance the load-bearing capacity, but they will inevitably lead to an increase in both costs and structural dead weight. In accordance with China’s “Code for Design of Concrete Structures” (GB/T50010-2010) [22], after a comprehensive consideration of various influencing factors, the thickness parameters of the composite floor specified in this paper are determined as follows: the thickness of straw boards ranges from 20 to 40 mm, and the thickness of concrete boards ranges from 60 to 80 mm.

**Figure 7:**
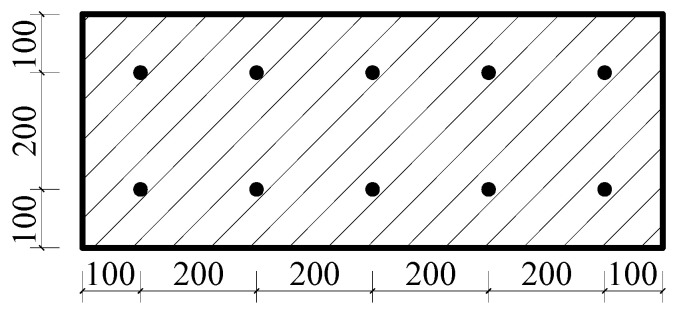
Connector arrangement for LB-A-1.

**Figure 8:**
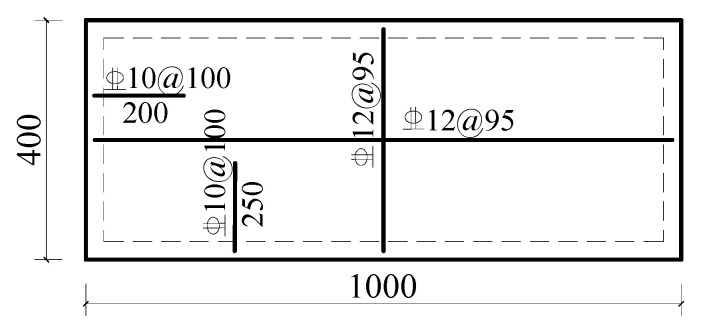
Reinforcement arrangement for LB-A-1.

The specific parameters are shown in Table 2. LB-A-1 was the control model. For this model, the thickness of the straw board was 20 mm, the thickness of the concrete was 70 mm, the size of the floor slab was 1000 × 400 mm, and the strength of the concrete was C30 according to the European “Code for the Design of Wood Structures” [24]. According to the principle of the arrangement of shear-resistant connectors in a wood structure, 10 self-tapping screws with a spacing of 200 mm were set. The “Code for the Design of Concrete Structures” (GB50010-2010) [22] was used to determine the diameter and spacing of the reinforcement connectors. The reinforcement arrangement is shown in Figure 7 and Figure 8. Since the reinforcement rate was not taken as a research parameter in this research, to prevent the reinforcement rate from influencing the finite element simulation results, the reinforcement was carried out in accordance with the same reinforcement method for combined floor slabs and concrete slabs with different thicknesses.

When designing the aforementioned composite floor slab model and determining its parameters, it is crucial to comprehensively consider the mechanical performance characteristics of the straw board and concrete. Specifically, the position of the neutral axis should be controlled within the concrete slab. Additionally, the compressive strength of the straw board should be fully exploited; meanwhile, the tensile reinforcement within the concrete slab should be utilized to bear the tensile forces.

## 3. Finite Element Model Establishment

**Figure 9:**
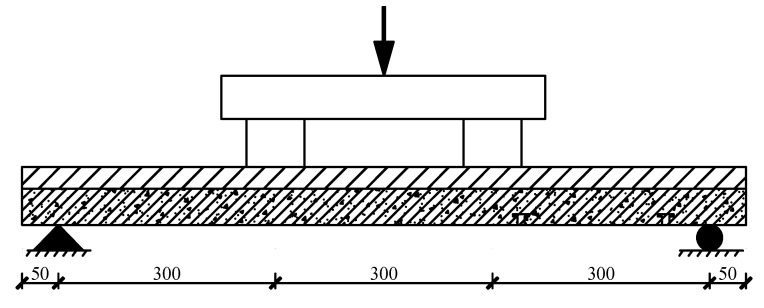
Simulated loading diagram of combined floor slabs/mm.

The flexural performance of straw–concrete combined floor slabs under loading was simulated using the ABAQUS finite element software. The simulated loading conditions are presented in Figure 9.

### 3.1. Finite Element Simulation Parameters

According to the test results of straw board materials, the orthotropic linear elastic model is adopted in ABAQUS software, version. The material parameters of the straw board are shown in Table 3.

The calculation formula is as follows:

(10) $$G12=\frac{E1}{2Nu12}$$

(11) $$G13=\frac{E2}{2Nu13}$$

(12) $$G23=\frac{E3}{2Nu23}$$

In the formula, G12, G13, G23 are the shear elastic modulus of the three planes; E1 is the tensile modulus of elasticity for the straw board in the X direction, E2 is the tensile modulus of elasticity of the straw board in the Y direction, and E3 is the compressive modulus of elasticity of the straw board in the Z direction; Nu12, Nu13, Nu23 are the Poisson’s ratios of the straw board, which are obtained through the material property experiment on the straw board.

The concrete strength grade is C30. In the ABAQUS software, the Concrete Damaged Plasticity is selected for simulation. The plastic damage parameters of C30 concrete are shown in Table 4.

The reinforcing steel in the study is HRB400, (Wuxi Tenide Metal Technology Co., Ltd., Wuxi, China). Considering the mechanical behavior characteristics of HRB400 steel and the need for accurate simulation in the analysis process, the double-slash model is utilized in the ABAQUS software for simulation. This model can effectively represent the stress–strain relationship of the tensile reinforcement under different loading conditions. The relevant parameters, which play a vital role in ensuring the accuracy of the simulation, are presented in Table 5.

In the ABAQUS software, the connector is simulated using the ideal elasto-plastic model. The relevant parameters are shown in Table 6.

### 3.2. Selection of Unit Types

To ensure the accuracy and precision of the simulation, the concrete slabs, straw slabs, bottom supports, and distribution beams were selected to be simulated with three-dimensional deformable reduced-integral solid cells (C3D8R). This is a cell type with excellent geometrical deformability and stress analysis capability and is particularly suitable for the simulation of complex geometries and deformation of solid structures. The connectors and force reinforcement were simulated with a 3D truss unit (T3D2). This is a lightweight and efficient unit type suitable for the linear analysis and simulation of axially stressed members, and it can optimize the overall use of computational resources.

### 3.3. Grid Division

An excessively fine mesh has the potential to result in disproportionately long computation times. Consequently, it is imperative to strike a balance between the desired accuracy of the simulation and the available computational resources. Drawing upon previous experience, implementing a structured mesh partitioning approach and setting the mesh sizes of both the concrete and straw board components at 15 mm is found to be more effective in simulating the internal stress distribution within these plates. Finer meshes possess the capability to more precisely capture the gradient of variations in the internal material stresses, thereby offering a closer approximation to the actual stress field. This also mitigates the occurrence of the “stress jumping” phenomenon typically associated with coarser meshes, ultimately enhancing the numerical precision of the simulation.

For connectors and load-bearing reinforcement elements within composite floor slabs, the mesh partitioning size is designated as 10 mm. This finer mesh size enables a more accurate simulation of the stress states in regions prone to stress concentration. Moreover, it allows for a more detailed tracking of the stress transfer process from the reinforcement to the concrete, providing more precise information regarding peak stress values and the stress distribution pattern. Such detailed information significantly contributes to improving the overall precision of the numerical simulation.

**Figure 10:**
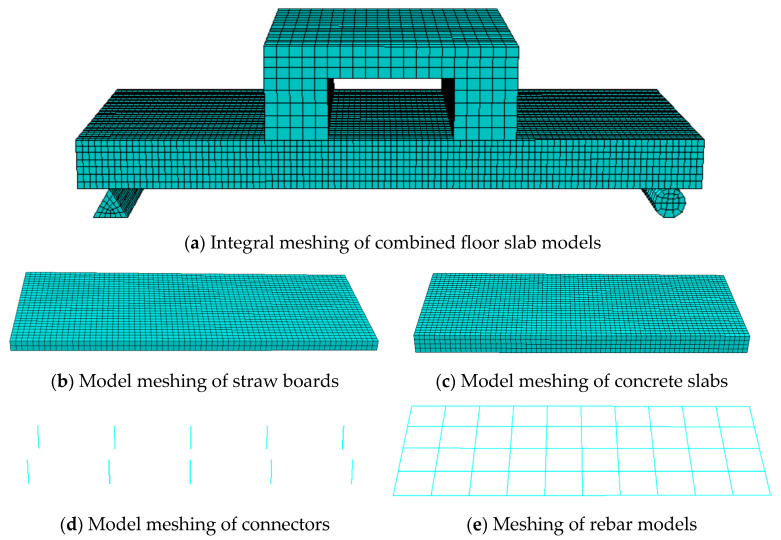
Meshing of the force model.

In the present simulation, as the deformation of the bottom supports and distribution beams is not taken into consideration, these components, namely the distribution beams and supports, are modeled as rigid bodies with a mesh size of 50 mm. Given that the specific details of their deformation are of no concern, a relatively coarse mesh suffices to describe their fundamental mechanical behavior. This approach not only simplifies the model but also substantially reduces the computational load and the time required for the simulation. The specific mesh partitioning scenario is illustrated in Figure 10.

### 3.4. Interface Interaction and Loading Methods

**Figure 11:**
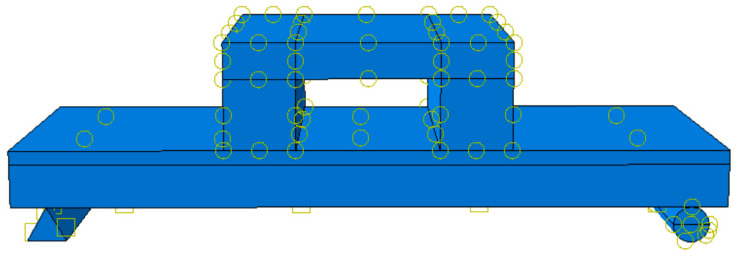
Interaction settings diagram.

When employing the ABAQUS finite element software, to integrate all components into a unified system and enable effective load transfer, constraint conditions are imposed at the interfaces of components based on their actual mechanical characteristics. Additionally, the contact relationships among the cross-sections of different components are defined. These contact relationships encompass the contact between the distribution beam and the straw board, the contact between the support and the concrete, the contact between the straw board and the concrete, the contact between the stressed reinforcement and the concrete, and the contact between connectors. Among these, the interactions between the upper distribution beam and the straw board, as well as between the support and the concrete, are set as face-to-face finite slip. In the normal direction, a “hard contact” condition is specified; for the tangential direction, the penalty friction formula is adopted, with the friction coefficient set to 0.2, as illustrated in Figure 11.

**Figure 12:**
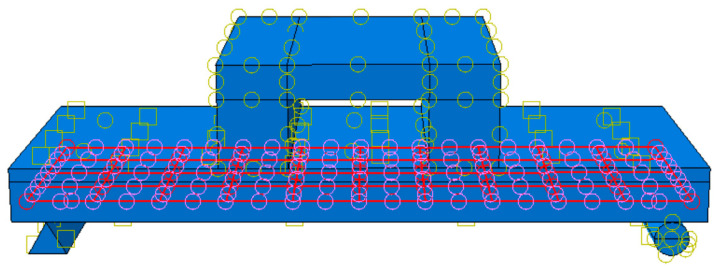
Reinforcing steel bar internal area.

**Figure 13:**
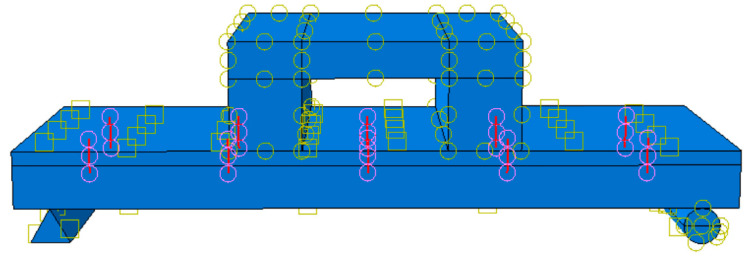
Connector internal area.

In the analysis, both the distribution beam and the supports are modeled as rigid bodies. A reference point, denoted as RP1, is established at the top of the distribution beam. Additionally, reference points RP2 and RP3 are set up at the two ends of the supports, respectively. Rigid body constraints are then imposed to couple the distribution beam and the supports to their corresponding reference points. To effectively simulate the bonding effect among the connectors, rebars, and concrete, the connectors and rebars are assigned a contact type of “internal-zone-constrained”. In this context, the internal zone encompasses the connectors and the stressed rebars, while the main zone is defined as the composite floor slab, as vividly illustrated in Figure 12 and Figure 13.

**Figure 14:**
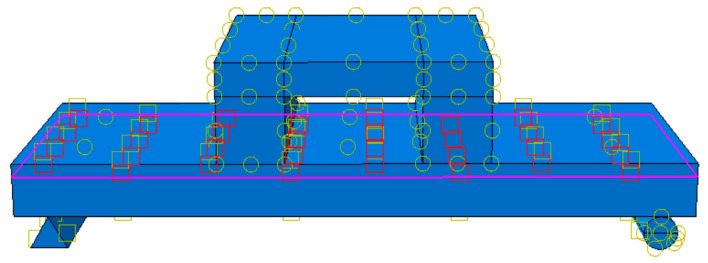
Cohesive contact between straw board and concrete.

In the analysis, for the bonded connection of composite floor slabs, the Cohesive model is chosen to attain the bonding effect. In ABAQUS, there exist two approaches to implementing the Cohesive model: one is through the use of Cohesive elements, and the other is via Cohesive contact. Among these, Cohesive elements offer a more extensive selection of constitutive relations. Nevertheless, during the simulation process, in the event that interface failure occurs, re-bonding cannot be accomplished. Additionally, the utilization of Cohesive elements will lead to an increase in the number of calculation analysis steps and an extension of the analysis time. Conversely, Cohesive contact defines the contact properties of the contact surface and has the capability to perform bonding at any time node, thereby enabling the simulation of secondary bonding. Simultaneously, it demonstrates greater advantages in terms of mesh size and convergence. Specifically, it can ensure a stable time increment and a consistent number of analysis steps. In this paper, the Cohesive contact method is selected to realize the bonding effect, and varying bonding strengths of the adhesive layers are achieved by altering the adhesive strength and fracture energy of the Cohesive contact, as depicted in Figure 14.

**Figure 15:**
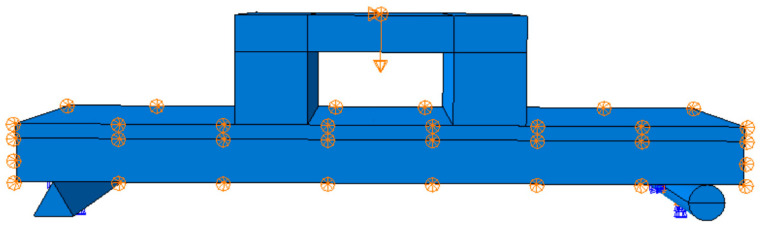
Load-setting diagram.

In the analysis, at the reference point RP2, the degrees of freedom are set as U1 = 0, U2 = 0, U3 = 0, UR1 = 0, UR2 = 0, and UR3 = 0 to simulate a fixed hinge support. This configuration restricts all translational and rotational movements at the reference point, mimicking the behavior of a fixed hinge in practical engineering scenarios. At RP3, the degrees of freedom are specified as U1 = 0, U2 = 0, and U3 = 0 to model a sliding hinge support, which allows for certain rotational movements while constraining the translational displacements in the corresponding directions. During the calculation process, a displacement loading method is employed. This approach is advantageous as it is more straightforward to couple with the structural model. The load is applied in a direction perpendicular to the composite floor slab, and it is exerted at the reference point RP1. This loading configuration is illustrated in Figure 15, providing a clear visual representation of the applied boundary conditions and loading setup for the analysis.

## 4. Finite Element Simulation Data Analysis

### 4.1. Analysis of Bending Moment-Deflection Relationship Curves

**Figure 16:**
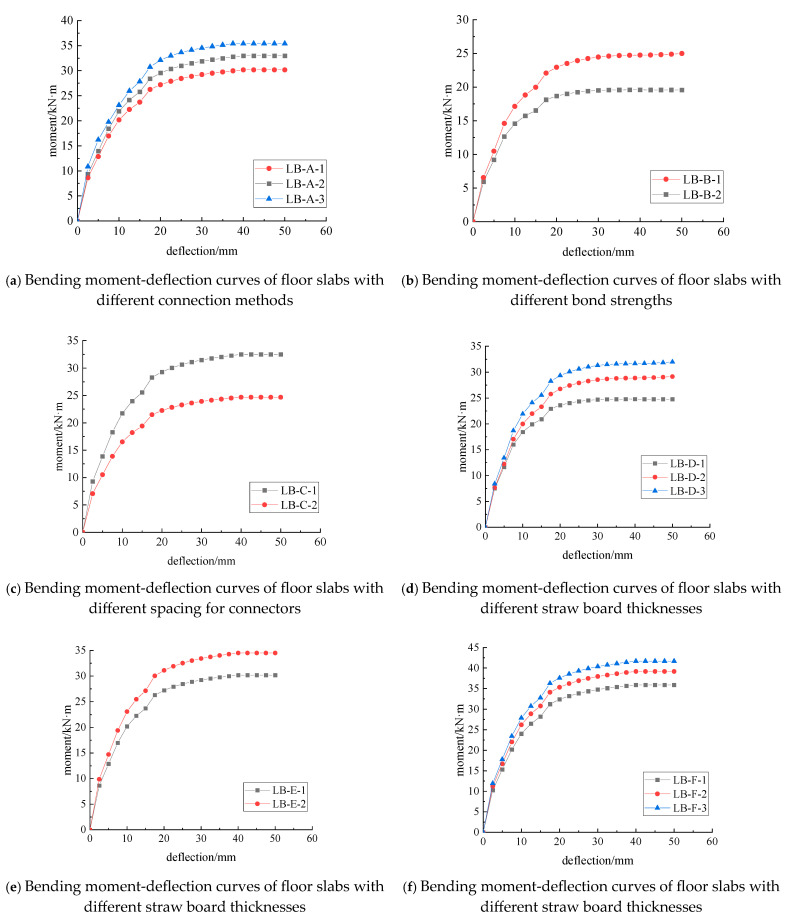
Moment-deflection curves of combined floor slabs.

The moment–midspan deflection curves of the straw–concrete combined floor slabs are shown in Figure 16.

It is evident from the figure that as the load increases, the deflection values of different composite floor slab models exhibit similar trends of development. In the initial stage of loading, the composite floor slab operates in the linear elastic working stage. At this juncture, the slope of the curve is steep, the mid-span deflection increases rapidly, and the stiffness of the composite floor slab gradually escalates. This phenomenon occurs because, prior to cracking, the interaction between the straw board and the concrete board is favorable. The stressed reinforcement and the concrete can jointly bear the tensile stress. The straw board possesses certain toughness and a relatively low mass, enabling it to work in synergy with the concrete during the elastic stage. Meanwhile, the concrete has a relatively high compressive strength, and the combined effect of these two materials endows the composite floor slab with excellent performance in the initial stage. In this stage, assuming that the materials are all in an ideal elastic state, the stress–strain relationship adheres to Hooke’s law, which is in good agreement with the actual behavior.

Upon continuous loading, the rate of deflection growth accelerates, the slope of the curve decreases continuously, and the stiffness also diminishes steadily. Consequently, the composite floor slab enters the elasto-plastic stage. This is attributable to the cracking of the concrete in the tensile zone at the bottom of the slab. The stressed reinforcement then takes over from the concrete to bear part of the tensile stress. As the position of the neutral axis shifts upward, the speed of crack propagation accelerates, and the stressed reinforcement enters the yield stage first. At this moment, cracks also appear in the straw board within the top compressed zone, yet it remains in the elastic working state, and the overall performance of the straw–concrete composite floor slab deteriorates. At this point, it is necessary to consider the nonlinear characteristics of the materials. After the concrete cracks, its tensile strength drops precipitously, and once the reinforcement enters the yield stage, its stress–strain relationship is no longer linear. These alterations in material characteristics have a crucial influence on the mechanical properties of the composite floor slab.

When the load reaches the ultimate load, the straw board at the top of the slab can effectively exert its compressive characteristics. Eventually, it reaches the ultimate compressive strain and is crushed. The reinforcement has already yielded completely, and the composite floor slab loses its bearing capacity. This indicates that the straw board and the stressed reinforcement jointly govern the flexural bearing capacity of the composite floor slab. The straw board can collaborate with the reinforcement to resist external forces.

Throughout the entire process, the moment-deflection curve of LB-A-3 consistently remains above those of LB-A-1 and LB-A-2, as depicted in Figure 16a. This suggests that bolts and self-tapping screws, as shear connection components, disrupt the overall stability of the composite floor slab. They are less effective than adhesive connections in providing more uniform connection forces. The ways in which bolts and self-tapping screws combine with the straw board and the concrete board differ from those of adhesives. Their connection points are relatively concentrated, making them prone to the occurrence of stress concentration phenomena and affecting the overall stability. In contrast, adhesives can be more evenly distributed on the connection interface and offer more stable connection forces.

When adhesive connection is employed, the curves of LB-B-1 and LB-B-2 exhibit a relatively high degree of coincidence in the initial stage of loading, as shown in Figure 16b. However, there are significant disparities in the subsequent loading stage. This indicates that a higher bonding strength can remarkably enhance the connection effect between the straw board and the concrete board, enabling them to jointly bear higher loads, transfer stress more effectively, and work in harmony.

From the start to the end of the loading process, the curves of LB-C-1 and LB-C-2 never show a tendency to converge, as illustrated in Figure 16c. This reveals that the spacing of the connection components continuously affects the load transfer mechanism at the contact interface between the two materials. Different spacings of the connection components will alter the stress distribution at the material contact interface. If the spacing is too small, stress concentration may arise, and if it is too large, the load cannot be transferred effectively. In the simulation, assuming different spacings of the connection components has a substantial impact on the simulation results.

When self-tapping nail connection is utilized, the moment-deflection curves of the models display similar trends of development, as shown in Figure 16d–f. This indicates that increasing the thickness of either the straw board or the concrete board can enhance the overall stiffness of the composite floor slab and improve the specimen’s anti-deformation ability, without significantly altering its failure mode. In the numerical simulation, after changing the plate thickness parameter, the mechanical property analysis of the model also validates this actual performance.

It can also be observed from Figure 16 that, under the condition of the same thickness of the straw board and the concrete, adhesive connection can bear the maximum ultimate load. Moreover, the greater the bonding strength and the smaller the spacing of the connection components, the larger the ultimate load that can be borne. Under the condition of the same thickness of the concrete board, the thicker the straw board, the greater the ultimate load it can bear. Additionally, the greater the thickness of both the straw board and the concrete board, the larger the ultimate load that can be borne.

### 4.2. Analysis of Parameters Affecting Flexural Properties

#### 4.2.1. Connection Methods

From the bending moment-deflection curve of the combined floor slab with different connection methods, it can be seen that at the early stage of loading, the curve shows a linear trend. The combined floor slab was in the elastic deformation stage, and the connection method had less influence on the bearing capacity of the combined floor slab. With the increase in the load, the bearing capacity of the combined floor slab with different connection methods began to diverge. The flexural load-bearing capacity of the combined floor slabs with different connection methods is listed in Table 7. From the table, it can be seen that the highest load-bearing capacity of the composite floor slab with adhesive bonding LB-A-3 was 7.34%, and 17.34% higher than those of the bolted and self-tapping nailed combined floor slabs, while the load-bearing capacity of the bolted combined floor slabs was 9.32% higher than that of the self-tapping screw combined floor slabs. This could be determined from the analysis because the adhesive bonding could achieve a larger connection area and adapt to the irregular shape of the connection interface and poor surface treatment, thus providing a more uniform deformation. The bolt connection and self-tapping screw connection would produce a stress concentration in the connection area, thus affecting the connection effect. It could be seen that compared with the bolt connection and self-tapping screw connection, the combined floor slab with adhesive bonding had the best integrity and the greatest load-bearing capacity.

#### 4.2.2. Bond Strength

The flexural bearing capacities of the combined floor slabs with different bond strengths are listed in Table 8. As can be observed from the table, the effect of the bond strength on the bearing capacity of the combined floor slabs was very obvious. When the bond strength increased from 40 MPa to 50 MPa, the bearing capacity of the combined floor slabs increased by 27.64%. When the bond strength increased from 50 MPa to 60 MPa, the bearing capacity of the combined floor slabs increased by 41.67%. It could also be seen that increasing the bond strength could enhance the shear performance of the contact surface of the straw board and concrete board, effectively transfer the load, reduce the relative sliding, improve the combination effect, and thus enhance the stiffness and stability of the whole combined floor slab, which in turn significantly improved its load-carrying capacity.

#### 4.2.3. Connector Spacing

The values of the flexural load-bearing capacity of the combined floor slabs with different connector spacings are listed in Table 9. As can be observed from the table, the connector spacing was inversely proportional to the load-bearing capacity. When the spacing of connectors was reduced from 400 mm to 200 mm, the bearing capacity of the combined floor slab increased by 22.25%. When the spacing was reduced from 200 mm to 160 mm, the bearing capacity of the combined floor slab increased by 7.79%. This was because the smaller spacing of the connectors could improve the degree of connection between the two materials and increase the effective contact area between the straw board and concrete. This enhanced the load transfer efficiency, effectively reducing the local stress concentration phenomenon and improving the overall stability and the flexural load-bearing capacity of the combined floor slab.

#### 4.2.4. Thickness of Straw Boards

**Figure 17:**
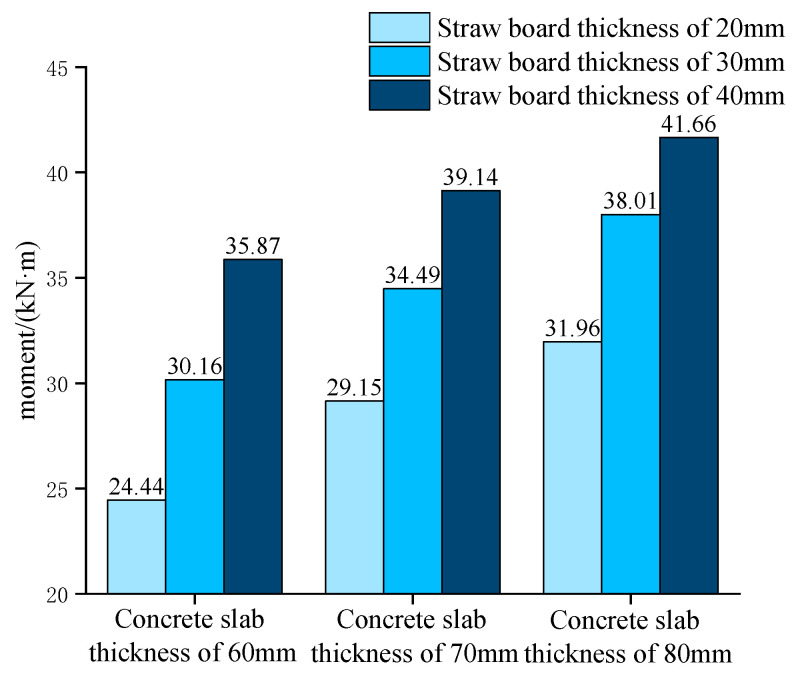
Flexural capacity of the combined floor slabs with different straw board thicknesses.

The flexural capacity of the combined floor slabs with different straw board thicknesses when the concrete slab thicknesses are 60 mm, 70 mm, and 80 mm, respectively, is shown in Figure 17.

The figure reveals that when the thickness of the concrete slab was certain, increasing the thickness of the straw plate could improve the ultimate load-carrying capacity of the straw–concrete combined floor slab. However, with the increase in the straw plate thickness, the growth of load-carrying capacity gradually decreased. The analysis revealed that this was because the greater the thickness of the straw board was, the more straw fibers there were, and the greater the moment of inertia of the cross-section was, which improved the overall flexural rigidity of the combined floor slab and could better resist the deflection caused by the load. However, as the thickness of the straw board increased further, the internal stress distribution of the straw board was not uniform, which limited the growth of the overall load-carrying capacity. The increased plate thickness increased the self-weight of the members, which led to a decrease in the shear strength and bond properties of the combined floor slabs, thus reducing the growth of the flexural load-carrying capacity.

#### 4.2.5. Concrete Slab Thickness

**Figure 18:**
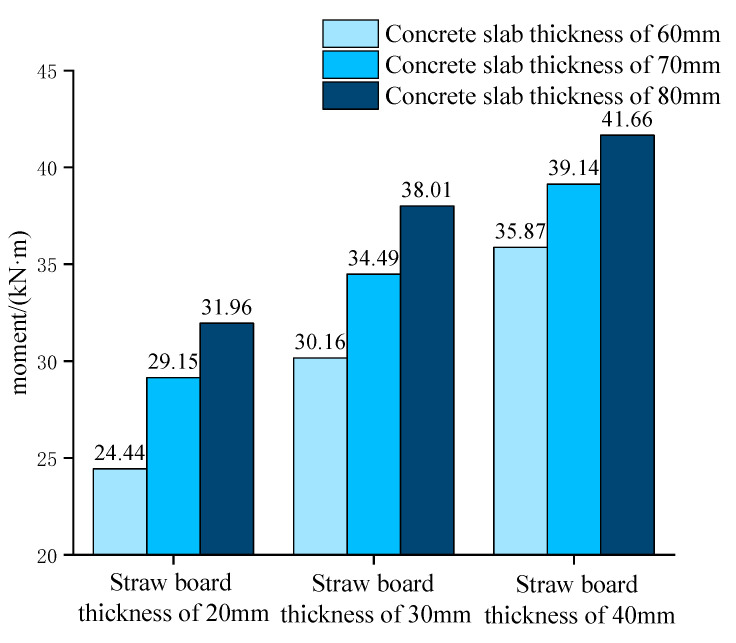
Flexural capacity of the combined floor slabs with different concrete slab thicknesses.

The flexural capacity of the combined floor slabs with different concrete slab thicknesses when the straw board thicknesses are 20 mm, 30 mm, and 40 mm, respectively, is shown in Figure 18.

The figure reveals that when the thickness of the straw slab was certain, increasing the thickness of the concrete slab could significantly improve the ultimate load-carrying capacity of the straw–concrete combined floor slab, and the improvement effect was more obvious than that of increasing the thickness of the straw slab. This was because the strength and stiffness of the concrete were better than that of the straw, and the increase in the thickness of the concrete slab could effectively reduce the deflection and deformation of the combined floor slab, which significantly improved the overall bearing capacity of the combined floor slab. However, as the thickness of the concrete slab increased further, the bond between the straw slab and the concrete slab was not strong enough to provide a reliable connection performance; therefore, the increase in the load-carrying capacity of the combined floor slab gradually decreased. In practical applications, the thickness of both the straw and concrete slabs should not be too large based on the consideration of both material usage and load-carrying capacity.

### 4.3. Analysis of Floor Damage Patterns

**Figure 19:**
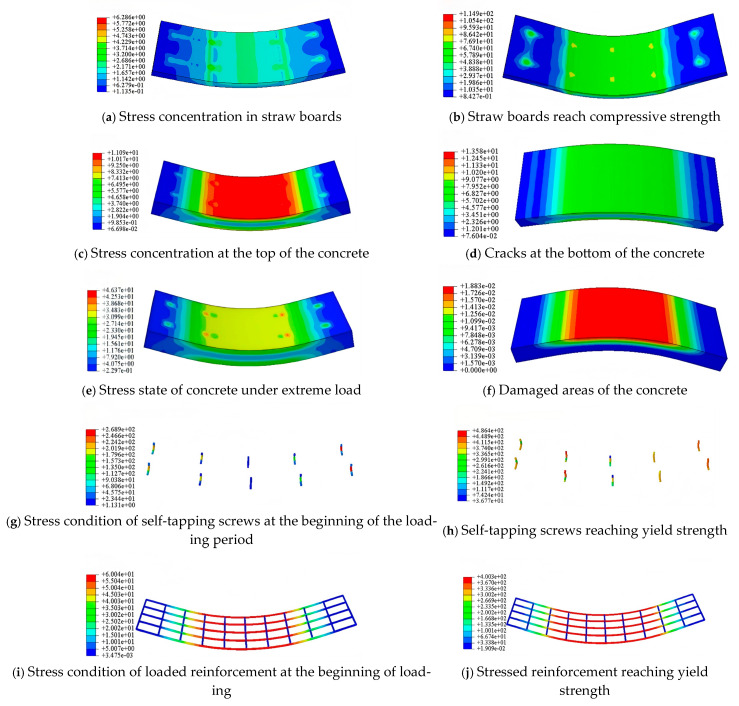
Stress cloud of a combined floor slab model.

The Mises stress cloud diagrams of the straw–concrete combined floor slabs were viewed and extracted in the visualization module of ABAQUS, as shown in Figure 19. The analysis of the damage process showed that the damage patterns of the combined floor slabs were relatively similar, and the main forms of damage were bending damage and longitudinal shear damage. In the 15 combined floor slab models, specimens LB-A-1, LB-A-2, and LB-A-3 experienced the bending damage mode. In the loading process, due to the low tensile strength of the concrete, the concrete at the bottom of the slab first had small cracks. The position of the neutral axis at this stage was slightly upward shifted compared with that at the beginning of the loading, and the height of the pressure zone gradually decreased. With the expansion of the concrete cracking area, the tensile strain at the original neutral axis position increased continuously, and the tensile reinforcement at the bottom of the combined floor slab was the first to yield. Then the straw plate at the top pressurized area reached the ultimate compressive strain to be crushed and lost the bearing capacity, which was categorized as the ductile bending damage mode. The straw board and concrete of specimen LB-A-3 were attached using adhesive, and the adhesive strength was large. The adhesive force restricted the relative slip between the interfaces of the combined floor slabs during the stressing process, and the overall deformation performance of the combined floor slabs was better, so the material properties of the straw boards and the stressed reinforcement bars could be fully used.

The damage patterns of specimens LB-B-1, LB-B-2, LB-C-1, and LB-C-2 were basically the same as those of LB-A-1, LB-A-2, and LB-A-3. However, due to the larger spacing of the connectors arranged in specimen LB-C-2, longitudinal shear damage occurred during the stressing process, and most of the self-tapping screws underwent yielding damage when the combined floor slabs had not yet reached the ultimate load-bearing capacity. This caused the effect of the combination of the concrete and straw boards to be gradually weakened, and a relative slip occurred between the interface of the materials.

The damage modes of specimens LB-D-1, LB-D-2, LB-D-3, LB-E-1, LB-E-2, LB-F-1, LB-F-2, and LB-F-3 also had similarities. As the thickness of the straw boards increased, the flexural capacity of the combined floor slabs became greater, and the strain growth of the stress reinforcement in the slabs decreased, with the yielding time being relatively late, showing better plastic deformation characteristics.

The typical damage pattern of the specimen could be divided into the following three stages:

① Linear elasticity stage: In this stage, the straw and concrete boards achieved a better combination effect through the connectors and jointly bore the load transmitted from the loading point. At this time, the maximum stress at the top of the straw board was at the loading point and the midspan position, as shown in Figure 19a. The maximum stress of the concrete slab occurred at the midspan position, as shown in Figure 19c. The connectors were subjected to a smaller load, and the maximum stress occurred at the height of the connecting surfaces of the straw board and the concrete slab. The nearer to the midspan position the location was, the smaller the stress of the connectors was, as shown in Figure 19g. The loaded reinforcement was subjected to the tensile force, and the value of the stress gradually decreased, with the midspan region extending to the end, as shown in Figure 19h. The stress value was reduced as the midspan region extended to the end. The region that extended to the end gradually decreased, as shown in Figure 19i. At this time, the midspan deflection of the combined floor slab increased with the increase in the load.

② Elastic–plastic stage: After the bottom of the concrete reached the tensile strength, the curve entered the elastic–plastic stage. The concrete slab entered the working state with cracks, and the neutral axis of the combined floor slab moved upward, as shown in Figure 19d. The end connectors gradually reached the yield strength, the connecting effect of the straw and concrete boards was weakened, and the end of the straw–concrete combined floor slab slipped. As the concrete at the bottom reached the tensile strength and stopped working, the stress value of the stress reinforcement spanning the middle position gradually increased to reach the yield strength. With the increase in the load, the yield region kept expanding, as shown in Figure 19e.

③ Load falling stage: When the top of the straw board reached the compressive strength and was crushed, the stress of the straw board decreased step by step from the midspan position to the end, and the stress value at the top of the straw board where the self-tapping screws were connected was larger, as shown in Figure 19b. The concrete compression zone reached the compressive strength at the midspan position, the concrete at the bottom cracked and failed, and the damaged area was gradually narrowed upward from the bottom of the concrete, as shown in Figure 19f. The end connectors had completely yielded and were damaged. There was a large deformation, as shown in Figure 19h. The stressed steel bar in the middle of the span position reached the yield strength, and the final destruction of the specimen occurred, as shown in Figure 19j.

### 4.4. Analysis of the Bending Moment-Strain Relationship Curves

**Figure 20:**
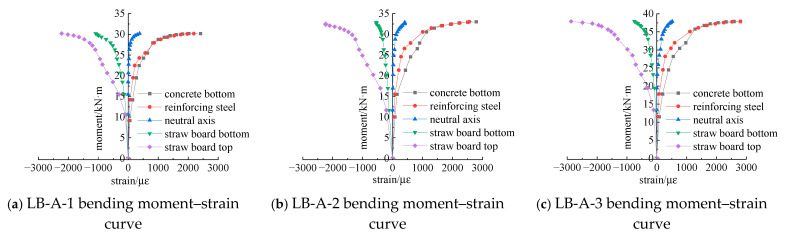
Bending moment–strain curve.

To analyze the strain variation at various heights within the dangerous cross-section structure of the straw board–concrete composite floor slab during the loading process, strain data from the bottom of the composite floor slab, the stressed reinforcement, the neutral axis, the bottom of the straw board, and the top of the straw board were extracted. Taking the mid-span cross-section of Group A as an illustrative example, the bending moment–strain relationship curves were plotted, as depicted in Figure 20.

By analyzing the curve trend in the figure, the flexural process of the composite floor slab can be divided into the following three stages:

(1) Uncracked working stage: At the initial loading stage, due to the small load, the stress state of the composite floor slab resembles that of a homogeneous elastic body. The tensile force is jointly borne by the tensile reinforcement and concrete in the tensile zone. The strain values of the bottom concrete and the tensile reinforcement are small, while the strain at the neutral axis position is zero. The straw board at the top of the slab is under compression. The load on each cross-section is proportional to the strain, the composite effect is good, and no cracking occurs in any component. In this stage, the composite floor slab behaves elastically, providing a stable foundation for subsequent loading.
(2) Cracked working stage: Subsequently, as the load continues to increase, the low tensile strength of concrete leads to the appearance of cracks in the bottom concrete, which manifests as a significant increase in strain. The tensile reinforcement then takes over part of the tensile stress from the bottom concrete. Consequently, the position of the neutral axis of the composite structure moves upward, and the height of the compressed zone gradually decreases. The tensile strain of the tensile reinforcement increases with the bending moment. Tensile strain also occurs at the original position of the neutral axis in the tensile zone, albeit with a small value. As the concrete cracking area extends towards the neutral axis, the tensile reinforcement enters the yield stage, and the tensile strain at the original neutral axis position continues to rise. The straw board at the top of the slab remains mostly in an elastic state. When cracks form in the straw board at the top of the composite floor slab, the compressive strain increases rapidly, and the slope of the curve decreases continuously. This stage marks the transition from elastic to inelastic behavior, with the development of cracks significantly affecting the structural performance.
(3) Yielding working stage: Eventually, when the ultimate load is reached, the straw board at the top reaches its ultimate compressive strain and is crushed. The stress nephogram shows that when the composite structure fails, the tensile reinforcement yields first, which is advantageous for fully utilizing the compressive properties of the straw board. The flexural bearing capacity of the composite floor slab structure is primarily determined by the straw board and the tensile reinforcement. At this stage, the composite floor slab loses its ability to withstand further loading, and the failure mechanism becomes dominant.

The trends of the moment–strain relationship curves for the other groups are analogous to those depicted in the above figure, and the failure modes of the composite floor slab structures are essentially identical. However, a more in-depth analysis of the data reveals that, within the composite structure, when the thicknesses of the straw board and the concrete are the same, the adhesive connection is capable of bearing the largest bending moment value and also exhibits the largest strain value. Furthermore, the greater the bonding strength and the smaller the spacing between the connectors, the larger the bending moment value and the strain value become. When the thickness of the concrete slab remains constant, the thicker the straw board, the larger the bending moment value and the strain value will be. Additionally, as the thicknesses of both the straw board and the concrete slab increase, the bending moment value and the strain value also increase accordingly.

### 4.5. Parameters for the Optimal Combination of Floor Slabs

To obtain the optimal parameter combination configuration of straw–concrete composite floor slabs, the end-slip values, ultimate moment values, maximum deflection values of each composite floor slab in the numerical simulation, along with their respective ratios, are tabulated in Table 10.

As is evident from Table 10, when the end-slip value of the composite floor slab structure is zero, it serves as the most favorable basis for parameter selection. In this case, the thickness of the straw board is 20 mm, and the thickness of the concrete is 70 mm. As previously discussed, the composite floor slab utilizing adhesive connection exhibits a relatively high load-bearing capacity. It is increased by 7.34% and 17.34% compared to the composite floor slabs connected by bolts and self-tapping screws, respectively. This is attributable to the fact that adhesive connection can achieve a larger connection area, resulting in uniform deformation and the best integrity of the composite floor slab.

The influence of the bonding strength on the load-bearing capacity of the composite floor slab is quite significant. When the bonding strength is increased from 40 MPa to 60 MPa, the load-bearing capacity of the composite floor slab is augmented by 80.84%. This is because enhancing the bonding strength can boost the shear resistance of the contact surface between the straw board and the concrete board. It enables the effective transfer of the load, reduces relative sliding, improves the composite effect, thereby enhancing the overall stiffness and stability of the composite floor slab, and subsequently leads to a remarkable increase in its load-bearing capacity.

The spacing of the connectors is inversely proportional to the load-bearing capacity. When the spacing of the connectors is decreased from 400 mm to 200 mm, the load-bearing capacity of the composite floor slab is elevated by 22.25%. This is due to the fact that a smaller connector spacing can enhance the connection tightness between the two materials. It increases the effective contact area between the straw board and the concrete, thereby improving the load-transfer efficiency, effectively reducing local stress concentration phenomena, and enhancing the overall stability and flexural load-bearing capacity of the composite floor slab.

When the thickness of the concrete board is held constant, as the thickness of the straw board increases, the growth rate of the load-bearing capacity is relatively modest. This is because the thicker the straw board, the more uneven the internal stress distribution within the straw board becomes, which restricts the growth of the overall load-bearing capacity. In practical applications, taking into account both material consumption and load-bearing capacity, the thicknesses of the straw board and the concrete board should not be excessively large.

In summary, by comprehensively considering the requirements of small end-slip, high flexural load-bearing capacity, and minimal deflection deformation, the optimal combination configuration of the composite floor slab is determined to be Model LB-A-3. Specifically, it refers to the composite floor slab with a straw board thickness of 20 mm, a concrete thickness of 70 mm, an adhesive connection method, a bonding strength of 60 MPa, and a connector spacing of 200 mm. This finding provides a valuable reference for practical engineering applications.

## 5. Theoretical Analysis

### 5.1. Calculation of Midspan Deflection

According to the Manual of Static Calculation of Building Structures [25], the midspan deflection of a straw–concrete combination floor slab is calculated as follows:

(13) $$f_{\max}=\frac{Pal^{2}}{24EI}\left(3-4\alpha^{2}\right)$$

In the formula, P is the load value corresponding to the elastic phase of the combined floor slab; a is the distance from the centralized load point to the support; l is the span of the combined floor slab; $\alpha =\frac{a}{l}$; EI is the flexural rigidity of the combined floor slab obtained with the converted cross-section method, $EI=E_{c}I_{\mathrm{eq}}=E_{c}\left(I_{w}+I_{s}+I_{c}\right)$, for which $E_{c}$ is the modulus of elasticity of the concrete; and $I_{w}$, $I_{s}$, and $I_{c}$ are the moments of inertia of the straw board, the tensile reinforcement, and the concrete slab on the shaped mandrel, respectively.

**Figure 21:**
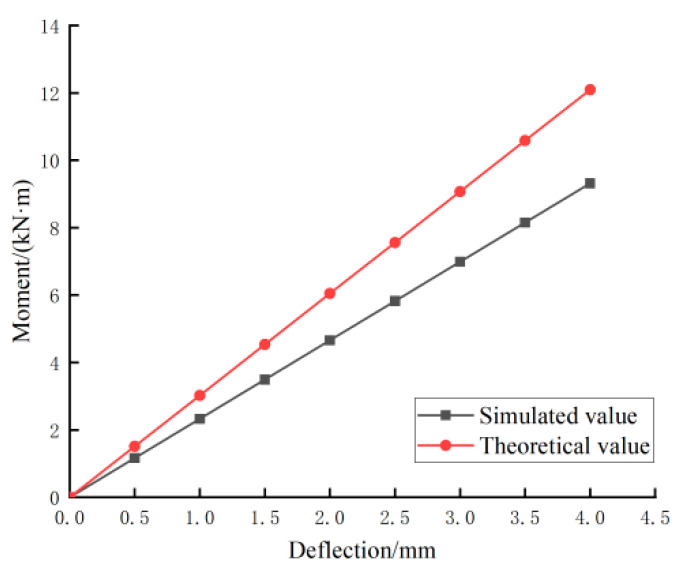
Comparison of simulated and theoretical values for model LB-D-1.

**Figure 22:**
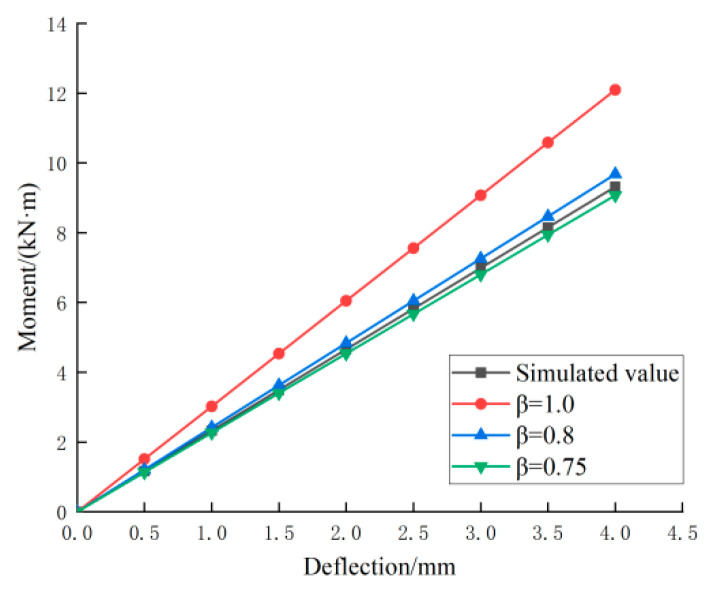
Load-deflection curves for different values of β.

The load–midspan deflection curves corresponding to the simulated and theoretically calculated values of member LB-D-1 in the normal use limit state are plotted in Figure 21. In the figure, it can be seen that the theoretical values were better than the simulated values. Therefore, with reference to the “Code for the Design of Concrete Structures”, the cross-section stiffness adjustment coefficient β was introduced to obtain the corrected midspan deflection formula of the combined floor slab during normal use, as shown in Equation (2). The load–midspan deflection curves corresponding to the theoretical values at different values of β are plotted in Figure 22. The figure indicates that when β was taken as 0.75, the theoretical curves were the closest to the simulated curves.

(14) $$f_{\max}=\frac{Pal^{2}}{24\beta EI}\left(3-4\alpha^{2}\right)$$

The theoretical and simulated load values for the allowable deflection are listed in Table 11 with reference to the Design Standard for Wood Structures [26], from which it can be seen that the error was less than 6% and that the theoretical and simulated values were in good agreement.

### 5.2. Calculation of Flexural Load Capacity

According to the Code for the Design of Concrete Structures, the theoretical analysis of the bending capacity of the straw–concrete composite floor slab at the load limit state was performed using the following assumptions:

(1) Both steel and concrete were regarded as ideal elastic materials with a direct proportional stress–strain relationship.
(2) No slip occurred at the interface between the straw board and the concrete slab during the entire process of force application.
(3) The strain distribution in the cross-section of the combined plate satisfied the flat cross-section assumption.
(4) The longitudinally distributed reinforcement within the concrete was negligible for positive bending moments.
(5) The tensile effect of straw boards in the tension zone was neglected.

**Figure 23:**
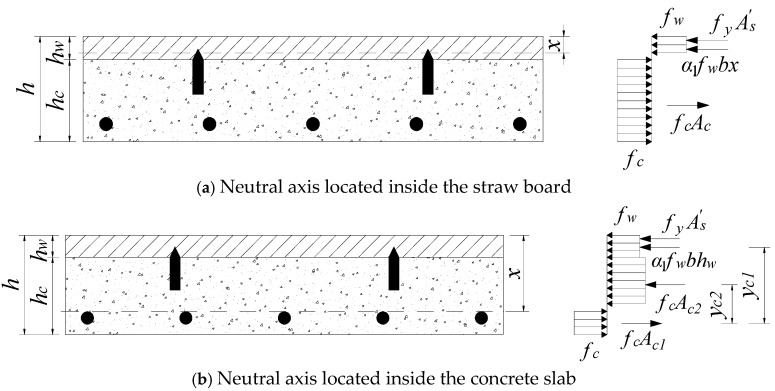
Calculation diagram.

The location of the neutral axis, when the load capacity limit state was reached, might have been within the straw slab or the concrete slab, as shown in Figure 23.

Depending on the location of the neutral axis, the bearing capacity of the combined floor slab could be calculated in the following two cases:

(4) When the neutral axis was located inside the straw slab, the full cross-section of the concrete slab was yielded in tension. The bending capacity of the straw–concrete combined floor slab is calculated as follows:

(15) $$\alpha_{1}f_{w}bx+f_{y}A_{s}^{\prime }=f_{c}A_{c}$$

(16) $$M\leq M_{u}=\alpha_{1}f_{w}bx(h_{0}-\frac{x}{2})+f_{y}A_{s}^{\prime }(h_{0}-a_{s}^{\prime })$$

where $M_{u}$ is the ultimate bending moment of the combined floor slab; $f_{c}$ is the design value of the compressive strength of the concrete; $f_{w}$ and $f_{y}$ are the yield strengths of the straw board and the compression reinforcement; and $A_{c}$ is the area of the concrete slab within the calculated width. Furthermore, $A_{s}^{\prime }$ is the cross-sectional area of the compression reinforcement, b is the calculated width of the combined floor slab, x is the height of the calculated compression zone, and $h_{0}$ is the effective height of the combined floor slab section. Additionally, $a_{s}^{\prime }$ is the distance from the center of compression reinforcement to the edge of the compression of the cross-section, and $\alpha_{1}$ is the coefficient of the concrete compression stress diagram.

(5) When the neutral axis was located inside the concrete slab, the neutral axis to the bottom surface of the combined floor slab part of the concrete was in a state of compression. However, the role of this part of the concrete is generally not considered. The straw–concrete combined floor slab bending capacity calculation formula is as follows:

(17) $$\alpha_{1}f_{w}bh_{w}+f_{y}A_{s}^{\prime }+f_{c}A_{c2}=f_{c}A_{c1}$$

(18) $$M\leq M_{u}=\alpha_{1}f_{w}bh_{w}y_{c1}+f_{c}A_{c2}y_{c2}+f_{y}A_{s}^{\prime }(y_{c1}+\frac{x}{2}-a_{s}^{\prime })$$

where $y_{c1}$ and $y_{c2}$ are the distances from the point of joint force in the tensile zone of the concrete slab to the point of joint force in the compression zone of the straw board and the point of the joint force in the compression zone of the concrete slab, respectively; $A_{c1}$ and $A_{c2}$ are the cross-sectional areas of the concrete slab below and above the neutral axis within the calculated width, respectively, and $A_{c1}+A_{c2}=A_{c}$.

The bending capacity under the ultimate state of composite slab can be calculated according to Equations (4) and (6). The theoretical and simulated values of the bending moment are listed in Table 12. As can be seen from the table, the error was less than 12%, which indicated that the cross-section load-bearing capacity formula could be a more accurate response to the real load-bearing capacity of the combined floor slab.

## 6. Conclusions

ABAQUS was used to perform finite element numerical analysis on six groups of 15 straw–concrete combined floor slabs to simulate the three-point static loading test and to study the damage mechanism and the factors affecting the flexural performance of the combined floor slabs. The following conclusions are drawn:

(1) Under the three-point loading condition, the damage mode of the combined floor slab was tensile cracking at the bottom of the concrete, and the stress reinforcement gradually yielded until the top of the straw plate reached the ultimate compressive strength and lost the bearing capacity. The overall damage to the combined floor slab was manifested as ductile bending damage. When the combined floor slab was subjected to a load, the overall working performance of the straw plate and concrete was good, and the deformation was coordinated to meet the assumption of a flat cross-section.

(2) Compared with the ultimate flexural load capacities of the bolted or self-tapping screw-connected composite floor slabs, those of the composite floor slab with adhesive bonding increased by 7.34% and 17.34%, respectively. For the same composite floor slab with adhesive bonding, when the bond strength was increased from 40 MPa to 60 MPa, the flexural load capacity increased by 80.84%. For the same self-tapping screw-connected combined floor slabs, when the distance of the connecting parts was reduced from 400 mm to 160 mm, the flexural load capacity increased by 31.78%. The increase in the thickness of the straw or concrete board had a significant effect on improving the load-carrying capacity of the combined floor slabs. Similarly to the self-tapping screw connection, when the spacing of the connectors was reduced from 400 mm to 160 mm, the bending capacity increased by 31.78%. The increase in the thickness of the straw or concrete board to enhance the bearing effect of the combined floor slab was obvious, for which the thickness of the concrete board had a greater effect on the ultimate bending capacity of the combined floor slab. However, with the further increase in the thickness, the increase in the load-bearing capacity of the combined floor slab gradually became smaller.

(3) After comprehensively considering the effects of the model parameters and connection methods on the overall performance of the straw–concrete combined floor slabs, the optimal parameter combination with a small end-slip, high bending capacity, and small deflection deformation was obtained, i.e., the parameter combination corresponding to the combined floor slab LB-A-3. It provides a reference basis for practical engineering applications.

(4) According to the converted section method and the static calculation formula for a building structure, the midspan deflection calculation formula and the flexural bearing capacity calculation formula in the limit state that applied to the normal use stage of the combined floor slab were proposed and compared with the finite element simulation data. The errors were within 15%, which verified the accuracy of the theoretical calculation results, providing a reference for the application of straw panels as a green building material in engineering.

## Figures and Tables

**Table 1:** Material mechanical property test specimen results.

Specimen Number	Tensile Strength/MPa	Compressive Strength/MPa	Tensile Modulus of Elasticity/MPa	Compressive Modulus of Elasticity/MPa
1	9.34	15.56	2576	3126
2	7.76	14.13	2585	2785
3	8.75	17.88	2647	2940
4	8.11	15.97	2723	3086
5	8.65	16.98	2813	2973
6	9.34	17.12	2597	2801
7	8.46	15.37	2646	2913
8	7.86	14.17	2416	3047
9	8.75	16.40	2632	3185
Standard Deviation	0.54	1.29	102	131
Average	8.56	15.95	2626	2984
Coefficient of Variation	6.32%	8.09%	3.90%	4.40%

**Table 2:** Modeling parameters for combined floor slab.

Group Number	Model Number	Straw Board Thickness/mm	Concrete Slab Thickness/mm	Connection Method	Bond Strength/MPa	Connector Spacing/mm
Group I	LB-A-1	20	70	Self-tapping screw	60	200
	LB-A-2	20	70	Bolt	60	200
	LB-A-3	20	70	Adhesive bonding	60	200
Group II	LB-B-1	20	70	Adhesive bonding	50	200
	LB-B-2	20	70	Adhesive bonding	40	200
Group III	LB-C-1	20	70	Self-tapping screw	-	160
	LB-C-2	20	70	Self-tapping screw		400
Group IV	LB-D-1	20	60	Self-tapping screw		200
	LB-D-2	30	60	Self-tapping screw		200
	LB-D-3	40	60	Self-tapping screw		200
Group V	LB-E-1	30	70	Self-tapping screw		200
	LB-E-2	40	70	Self-tapping screw		200
Group VI	LB-F-1	20	80	Self-tapping screw		200
	LB-F-2	30	80	Self-tapping screw		200
	LB-F-3	40	80	Self-tapping screw		200

**Table 3:** Parameters of straw board materials.

E1/MPa	E2/MPa	E3/MPa	Nu12	Nu13	Nu23	G12/MPa	G13/MPa	G23/MPa
2626	2984	2626	0.28	0.28	0.28	1025	1165	1025

**Table 4:** Plastic damage parameters of C30 concrete.

Dilation Angle/°	Eccentricity/%	$f_{b0}$ $/f_{c0}$	K	Viscosity Parameters/Pa·s
30	0.1	1.16	0.667	0.0005

**Table 5:** Mechanical properties of reinforcing steel materials.

Elastic Modulus/GPa	Nu	Yield Strength Standard Value/GPa
200	0.3	400

**Table 6:** Material parameters of connectors.

Materials	Yield Strength/MPa	Elastic Modulus/GPa
Self-tapping screw	400	200
Bolt	400	200
Adhesive	60	4

**Table 7:** Bearing capacity of combined floor slabs with different connections.

Model Number	Straw Board Thickness/mm	Concrete Slab Thickness/mm	Connection Method	Moment /(kN·m)	Growth Rate/%
LB-A-1	20	70	Self-tapping Screw	30.16	-
LB-A-2	20	70	Bolt	32.97	9.32
LB-A-3	20	70	Viscid	35.39	7.34

**Table 8:** Bearing capacity of combined floor slabs with different bond strengths.

Model Number	Straw Board Thickness/mm	Concrete Slab Thickness/mm	Bond Strength/MPa	Moment /(kN·m)	Growth Rate/%
LB-B-2	20	70	40	19.57	-
LB-B-1	20	70	50	24.98	27.64
LB-A-3	20	70	60	35.39	41.67

**Table 9:** Bearing capacity of combined floor slabs with different spacing for connectors.

Model Number	Straw Board Thickness/mm	Concrete Slab Thickness/mm	Spacing/mm	Moment /(kN·m)	Growth Rate/%
LB-C-2	20	70	400	24.67	-
LB-A-1	20	70	200	30.16	22.25%
LB-C-1	20	70	160	32.51	7.79%

**Table 10:** End-slip values, ultimate moment values, maximum deflection values, and their ratios for combined floor slabs.

Model Number	End-Slip/(mm)	Ultimate Moment/(kN·m)	Maximum Deflection/(mm)	M: ε Ratio
LB-A-1	4.51	30.16	30.14	1.00
LB-A-2	4.18	32.97	31.36	1.05
LB-A-3	0	35.39	32.48	1.09
LB-B-1	0	24.98	29.31	0.85
LB-B-2	0	19.57	28.67	0.68
LB-C-1	5.17	32.51	31.14	1.04
LB-C-2	4.21	24.67	29.61	0.83
LB-D-1	3.35	24.44	29.09	0.84
LB-D-2	3.81	29.15	30.41	0.96
LB-D-3	4.12	31.94	30.71	1.04
LB-E-1	5.17	34.49	32.58	1.06
LB-E-2	5.51	38.01	33.55	1.13
LB-F-1	5.24	35.87	32.92	1.09
LB-F-2	5.91	39.14	34.29	1.14
LB-F-3	6.31	41.66	35.14	1.19

**Table 11:** Theoretical and simulated load values of combined beams with the allowable deflection.

Model Number	Theoretical Load/(kN·m)	Simulated Load/(kN·m)	Error/%
LB-D-1	9.07	9.32	−2.76
LB-D-2	9.62	9.78	−1.66
LB-D-3	10.18	10.73	−5.40

**Table 12:** Theoretical and simulated load values of combined beams with allowable deflection.

Model Number	Theoretical Load/(kN·m)	Simulated Load/(kN·m)	Error/%
LB-D-1	26.62	24.44	+8.92
LB-D-2	32.36	29.15	+11.01
LB-D-3	34.81	31.96	+8.92

## Data Availability

The original data presented in this study are included in the article.
